# It is a family affair: individual experiences and sibling exposure to emotional, physical and sexual abuse and the impact on adult depressive symptoms

**DOI:** 10.1017/S0033291720000823

**Published:** 2021-09

**Authors:** Marie-Louise Kullberg, Charlotte van Schie, Eleonore van Sprang, Dominique Maciejewski, Catharina A. Hartman, Bert van Hemert, Brenda W. J. H. Penninx, Bernet M. Elzinga

**Affiliations:** 1 Institute of Clinical Psychology, Leiden University, Leiden, The Netherlands; 2 Leiden Institute for Brain and Cognition (LIBC), Leiden University Medical Center, Leiden, The Netherlands; 3 School of Psychology and Illawarra Health and Medical Research Institute, University of Wollongong, Wollongong, Australia; 4 Amsterdam UMC, Vrije Universiteit, Psychiatry, Amsterdam Public Health Research Institute, Amsterdam, The Netherlands; 5 Department of Developmental Psychopathology, Behavioral Science Institute, Radboud University, Nijmegen, The Netherlands; 6 Department of Psychiatry, University of Groningen, University Medical Center Groningen, Interdisciplinary Center Psychopathology and Emotion Regulation, Groningen, The Netherlands; 7 Department of Psychiatry, Leiden University Medical Center, Leiden, The Netherlands

**Keywords:** Abuse, childhood maltreatment, depression, Family, multilevel, neglect, siblings

## Abstract

**Background:**

Childhood abuse and neglect often occurs within families and can have a large influence on mental well-being across the lifespan. However, the sibling concordance of emotional abuse and neglect (i.e. together referred to as emotional maltreatment; EM), physical abuse (PA) and sexual abuse (SA) and the long-term impact on the context of siblings' maltreatment experiences are unclear. To examine the influence of EM, PA and SA on adult depressive symptoms within the family framework we differentiate between (a) the family-wide (mean level of all siblings) effects and (b) the individual deviation from the mean family level of maltreatment.

**Methods:**

The sample (*N* = 636) consists of 256 families, including at least one lifetime depressed or anxious individual and their siblings. Multilevel modeling was used to examine the family-wide and relative individual effects of childhood maltreatment (CM).

**Results:**

(a) Siblings showed most similarity in their reports of EM followed by PA. SA was mostly reported by one person within a family. In line with these observations, the mean family levels of EM and PA, but not SA, were associated with more depressive symptoms. In addition, (b) depression levels were more elevated in individuals reporting more EM than the family mean.

**Conclusions:**

Particularly in the case of more visible forms of CM, siblings' experiences of EM and PA are associated with the elevated levels of adult depressive symptoms. Findings implicate that in addition to individual maltreatment experiences, the context of siblings' experiences is another crucial risk factor for an individuals' adult depressive symptomatology.

## Introduction

Depression is one of the most prevalent mental health problems worldwide (De Graaf, Ten Have, Van Gool, & Van Dorsselaer, 2012; World Health Organization, 2017) and accounted for 40.5% of disability-adjusted life years (Whiteford et al., 2013). As most psychiatric disorders, depression finds its roots in adverse childhood circumstances like parental abusive behavior (Kessler et al., 2010; Norman et al., 2012). Exposure to childhood maltreatment (CM) is associated with an increased risk of mood disorders (Norman et al., 2012) and in particular depression (Spinhoven et al., 2010), as opposed to other psychopathology. Epidemiological studies have shown that emotional abuse and neglect (emotional maltreatment; EM) strongly predict depression (Cecil, Viding, Fearon, Glaser, & McCrory, 2017; Gerke et al., 2018), followed by sexual abuse (SA) and physical abuse (PA; Infurna et al., 2016; Spinhoven et al., 2010). The high prevalence of abuse and neglect across the population (Stoltenborgh, Bakermans-Kranenburg, Alink, & van Ijzendoorn, 2015) and its effect on emotional disorders across the entire lifespan (Ege, Messias, Thapa, & Krain, 2015; Nanni, Uher, & Danese, 2012) contributes to the fact that CM has been identified as one of the greatest psychopathology risk factors (Sara & Lappin, 2017).

### Similarities in sibling experience of CM

Studies on the impact of abuse and neglect and the association with psychopathology have typically focused on a variety of consequences of CM for individuals being at risk. However, CM typically involves families, and siblings of maltreated children are likely exposed to similar parental behaviors (Baldwin & Oliver, 1975; Hamilton-Giachritsis & Browne, 2005; Hines, Kantor, & Holt, 2006; Jean-Gilles & Crittenden, 1990). Studies have shown greatest overlap between siblings in reported emotional abuse and neglect, followed by PA and neglect, whereas SA seems to show the least overlap (MacMillan, Tanaka, Duku, Vaillancourt, & Boyle, 2013; Witte, Fegert, & Walper, 2018). The degree of concordance may be related to whether the perpetrator is a family member, since in the case of EM and PA the perpetrator is a parent in 80% of the cases, whereas in SA this is most often someone else (86%; Bifulco, Brown, Lillie, & Jarvis, 1997; Hovens *et al*. 2009). Although ~90% of the Western individuals grow up with at least one sibling (Milevsky, 2013) and siblings are at increased risk for similar maltreating behavior, until now the influence of any CM type on adult functioning is mostly studied in unrelated individuals, not considering the experiences of siblings growing up in the same household.

Whether and how siblings' childhood experiences of abuse and neglect are related to depressive symptomatology of an individual on the long-term remains unclear. To unravel the psychological consequences of CM in the family context, a differentiation needs to be made between the impact of family-wide (e.g. an adverse family atmosphere) and the individual experiences contrasting with those of other siblings (e.g. ‘being the black sheep’), within the family (Feaster, Brincks, Robbins, & Szapocznik, 2011; Steinglass, 1987).

### Family-wide effects of CM

Within a family context, when one sibling is exposed to abuse or neglect by a parent or other family members may also be at risk by being exposed to the same harmful behavior. In addition to direct exposure, emotional abuse (EA) such as criticism (e.g. ‘you are stupid or lazy, you are the most worthless child ever born’) or physical violence (e.g. getting hit or kicked by a parent), could also affect siblings indirect by being the spectator, i.e. the vicarious effect of abuse (Spano, 2018). Thus, a key question we aim to investigate is whether CM reported by siblings growing up together (i.e. the family level) relates to an individual's adult depression.

### The impact of being the black sheep

Research on negative parenting in children indicates that, next to CM experienced by all or several children in the family, receiving more parental negativity compared to other siblings (i.e. being the black sheep) is also associated with unfavorable mental health outcomes (Dunn, Stocker, & Plomin, 1990; Jenkins, McGowan, & Knafo-Noam, 2016; Meunier, Bisceglia, & Jenkins, 2012; Pike & Plomin, 1996). For example, children reporting less support from their parents relative to their sibling (less favored) reported more depressive symptoms in young adulthood (Jensen, Whiteman, Fingerman, & Birditt, 2013). Hence, the second question we aim to address is whether being more emotionally maltreated or physically or sexually abused than other siblings from the same family (i.e. being the black sheep) is related to adult depressive symptomatology.

### The present study

High concordance, and moreover an impactful family level of CM would imply that CM is engrained in the family structure and could point out the potential harm of siblings' childhood experiences of abuse and neglect on the long term. Studying the family-wide and relative effects of harsh parenting has shown to be insightful in children and adolescents (Jenkins et al., 2016). However, in adult siblings, the family context of CM and the long-term impact of EM, PA and SA on depressive symptomatology has, to the best of our knowledge, never been investigated. A within-family approach provides new insights into the family-wide impact of CM and the individual relative harm compared to other siblings that cannot be detected or understood with a between-subjects design, without the reports of siblings from the same household.

To elucidate the sibling concordance of abuse and neglect and evaluate the potential harm of CM within a family framework, we aim to: (1) examine the extent to which the reports on the three types of CM are similar among siblings (i.e. sibling concordance), where we expect to find most similarity for EM, followed by PA and SA; (2a) elucidate whether the family level of EM, PA and SA is associated with adult depressive symptoms; (2b) and elucidate whether the experience of being maltreated more (EM, PA and SA) relative to the other siblings in the family is associated with more depressive symptoms, while accounting for the family level of CM. Based on the abovementioned literature, it is hypothesized that family levels of CM are associated with individual depressive symptomatology and that the experience of being maltreated more than the other siblings is associated with more depressive symptoms with the strongest effects for EM.

## Method

The present study is part of the Netherlands Study of Depression and Anxiety (NESDA), an ongoing longitudinal cohort study started in 2004, aiming to determine the long-term course and consequences of depression and anxiety. A detailed description of the NESDA study design can be found elsewhere (Penninx et al., 2008). The study protocol was approved by the Ethical Review Board of Amsterdam Medical Centre, location VUmc and by local review boards of each participating center. After full verbal and written information about the study, written informed consent was obtained from all participants. At the 9-year follow-up (wave 6, 2014–2017), 380 siblings from 256 participants with a lifetime anxiety and/or depressive disorder were interviewed to collect data on anxiety and depression, psychosocial functioning and health (behavior) to examine the family context of the development of depression and anxiety disorders in this cohort. For the current study, information on CM and depressive symptoms was collected using self-report measures between 2010 and 2013, 6 years after baseline in wave 4 (W4) and at wave 6 (W6), 9 years after baseline. Siblings participated in W6 only. In the analyses, data of the affected targets collected in W4 and at W6 and sibling data from W6 were used.

### Sample

The study sample consisted of 636 participants, within 256 unique families. In total, 380 siblings participated, aged 20–78 years, with and without depression and anxiety disorders, who were related to 256 original NESDA participants with a lifetime anxiety and/or depressive disorder diagnosis, i.e. the affected targets. Inclusion criteria for affected targets were: (1) a lifetime anxiety and/or depressive disorder diagnosis assessed based on the CIDI psychiatric interview (see below) at least two time points during NESDA measurements; (2) 100% the same biological parents as their siblings; (3) participated in at least three out of four NESDA face-to-face interviews; (4) availability of genetic information; (5) approval of contacting siblings for research purposes and (6) participated in 9-year follow-up face-to-face interview. Inclusion criteria for the siblings were (1) currently living in the Netherlands; (2) aged between 18 and 78 years and (3) willing to participate in the 9-year follow-up (W6) face-to-face interview. Targets and siblings with a diagnosis of psychotic disorder, obsessive–compulsive disorder, bipolar disorder or severe addiction disorder were excluded. A second exclusion criterion was not being fluent in Dutch. Individual and family characteristics are described in Table 1. Mean age of respondents was 49.7 years, 62% was female. Of all respondents, 74% reported EM and 9% reported any form of PA. For SA, 18% from the respondents reported any experience of SA before the age of 16 based on the Childhood Trauma Questionnaire (CTQ). For more detailed information on the prevalence of CM in this sample see Table A1 and Fig. A1 from Appendix 1 in the online Supplementary materials. From 380 siblings, 191 (50.3%) were lifetime affected with an anxiety and/or depressive disorder and 189 siblings (49.7%) did not have a lifetime anxiety and/or depression.

**Table 1.** Sample characteristics of 636 respondents from 256 families

*Individual characteristics* (*N* = 636)	
Female, *N* (%)	397 (62.4)
Level of education	
Basic (lower vocational education), *N* (%)	14 (2.2)
Intermediate (higher vocational education), *N* (%)	318 (50.0)
High (college/university education), *N* (%)	304 (478)
Age (years), *M* (s.d.)	49.68 (13.2)
Lifetime anxiety disorder, *N* (%)	338 (53.1)
Lifetime depression, *N* (%)	376 (59.1)
Lifetime comorbid depression and anxiety, *N* (%)	267 (42.0)
Current depression, *N* (%)	65 (10.2)
Current anxiety disorder, *N* (%)	100 (15.7)
Depressive symptoms, *M* (s.d.)	14.53(10.4)
Childhood maltreatment total, *M* (s.d.)	37.88 (10.6)
Emotional maltreatment, *M* (s.d.)	9.82 (3.6)
Physical abuse, *M* (s.d.)	5.63 (1.8)
Sexual abuse, *M* (s.d.)	5.78 (2.4)
*Family characteristics* (*n* = 256)	*N* (%)
Number of participating individuals per family	
2	168 (65.6)
3	61 (23.8)
⩾4	27 (10.5)
Total family size	
2	82 (32.0)
3	73 (28.5)
4	42 (16.4)
5	23 (9.0)
6	21 (8.2)
⩾7	15 (5.9)
Sibling constellation	
Same sex, male	28 (10.9)
Same sex, female	92 (35.9)
Mixed sex	136 (53.1)
Maximum age difference	
0–5 years	147 (57.4)
6–9 years	75 (29.3)
10–19 years	34 (13.3)

### Measures

#### Psychopathology

*Inventory of Depressive Symptomatology-SR (IDS-SR)*: The Inventory of Depressive Symptomatology (IDS) is a self-report questionnaire designed to measure the number of depressive symptoms (Rush, Giles, & Schlesser, 1986). The questionnaire consists of 30 items, each with four answering options from 0 through 3. Sum scores on the items range from 0 to 84, with higher values indicating more symptoms of depression. The psychometric properties are acceptable; for instance, high correlations were found between the IDS and scores on the Hamilton Depression Rating Scale and Beck Depression Inventory (Rush, Gullion, Basco, Jarrett, & Trivedi, 1996). The IDS showed excellent internal consistency (*α* = 0.95) in the current sample. Depressive symptomatology was measured in W6 for all participants.

*Composite Interview Diagnostic Instrument (CIDI)*: The presence (current and lifetime) of DSM-IV-TR (American Psychiatric Association, 2000) based depressive disorders (dysthymia and major depressive disorder) and anxiety disorders (generalized anxiety disorder, social phobia, panic disorder with or without agoraphobia and agoraphobia) was established using Composite Interview Diagnostic Instrument (CIDI, version 2.1, WHO) in W6. The CIDI is used worldwide in clinical and epidemiological studies (e.g. de Graaf *et al*. 2010; Kessler *et al*. 2010) and high validity for depressive and anxiety disorders were found (Wittchen, 1994).

#### Childhood maltreatment

##### Childhood Trauma Questionnaire-Short Form (CTQ-SF)

The CTQ-SF is a 25-item retrospective questionnaire assessing five types of CM before the age of 16: emotional abuse (EA), physical abuse (PA) and sexual abuse (SA), as well as emotional neglect (EN) and physical neglect (PN). Each scale consists of five items scored on a 5-point Likert scale ranging from *never true* to *very often true*. A sum score on the CTQ, ranging from 25 to 125, is calculated by adding the five subscales. Data were collected during W4 for the targets and during W6 for the siblings. Psychometric properties were good (Bernstein et al., 2003; Spinhoven et al., 2014; Thombs, Bernstein, Lobbestael, & Arntz, 2009). The internal consistency of the CTQ (*α* = 0.88) and most subscales are excellent in the current sample (PA: *α* = 0.92; SA: *α* = 0.94; EA: *α* = 0.90; EN: *α* = 0.89). Because of the moderate internal consistency for PN (*α* = 0.45), this subscale is excluded from the analyses. Given the large overlap between EA and EN (*r* = 0.63, *p* < 0.001) we combined the EA and EN subscale into an EM subscale, in line with previous studies in the NESDA cohort by taking the average of the two subscales (see Van Der Werff *et al*. 2013; van Harmelen *et al*. 2010), for similar definition see the American Professional Society on the Abuse of Children (APSAC) and Glaser (2002). The Cronbach's *α* for the combined emotional abuse and neglect scale was 0.89 in our sample. Subscales used in the analyses are EM, PA and SA. Scores on the CTQ subscales can be assorted into four categories: no maltreatment (i.e. EM score 5–7, PA score 5–7, SA score 5), low maltreatment (i.e. EM 8–10; PA 8–9; SA 6–7), moderate maltreatment (i.e. EM 11–14; PA 10–12; SA 8–12) and severe maltreatment (i.e. EM > 14; PA > 13, SA > 13; Bernstein and Fink, 1998).

### Statistical analyses

#### Handling missing data

Data cleaning, preparation and descriptive statistics were performed with IBM SPSS Statistics 23.0 (SPSS Inc., Chicago, Illinois). For the IDS, 1.6% and for the CTQ 2.8% was missing and Little's Missing Completely at Random (MCAR) test indicated that data were not MCAR (χ^2^ = 16.23, df = 8, *p* = 0.039). As compared to those with complete data, those with missing data tend to be younger, *t*_(634)_ = −3.2, *p* ⩽ 0.001. There were no differences regarding gender, educational level, CTQ and IDS scores between those with complete and those with missing data (all *p*'s > 0.05). To retain the sample size, missing data were handled using multiple imputations (*mice-*package version 3.3.0; Van Buuren and Groothuis-Oudshoorn, 2011), carried out with R version 3.5.0 (R Core Team, 2018). A detailed description of the procedure of imputation can be found in the online Supplementary materials, Appendix 2. The newly generated datasets reflected the original means. The parameter estimates of all models were combined according to Rubin's (1987) rules.

#### Analyses

We first tested whether the previously found association between individual levels of EM, PA and SA and depressive symptoms (Hovens et al., 2012; Spinhoven et al., 2010) could be replicated in the current sample using a multilevel regression model with a grouping variable unique for each family as a random effect to control for the family structured data. Sum scores per CTQ subscale (EM, PA and SA) were taken as predictors, and IDS sum scores as outcome. Subsequently, to investigate the main research questions, first the sibling concordance of CM, i.e. the degree of similarity within the family for CM, was assessed using intraclass correlations (ICC) for EM, PA and SA (1). The ICC was calculated by dividing the total family variance by the between-family variance on the CTQ subscale, which gives an indication of the degree of sibling resemblance (Higgins & Keller, 1975; Shoukri & Ward, 1989). A high correlation within families is defined as a coefficient of 0.3 or higher (Donner, Eliasziw, & Shoukri, 1998). For the calculation of the ICC the *lme4-*package version 1.1-17 (Bates, Mächler, Bolker, & Walker, 2014) was used with R version 3.5.0 (R Core Team, 2018).

To identify the family-wide (common across all siblings) and relative (individual deviation from the siblings mean) effects of EM, PA and SA on depressive symptoms, regression analyses using multilevel modeling with two-levels were performed with a family grouping variable as random effect. First, an unconditional means model, without predictors, was built to calculate the ICC. In the baseline model age, gender and educational level were added as covariates. Model 1 includes the family means of the CTQ subscales as predictor variables to assess the contribution of the family level EM, PA and SA, common across all siblings, to individual depressive symptoms and examine between-family differences (2a). Next, in model 2, the individual deviations from the family mean were added to model 1 to identify the effect of the relative level of EM, PA and SA on depressive symptoms and examine within-family differences (2b). A description of the exact calculation of the predictors can be found in the online Supplementary materials, Appendix 3. Model fit was compared according to the methods for multilevel models with multiple imputed data described by Li, Meng, Raghunathan, and Rubin (1991) using the *F* test. Multilevel regression analyses (*lme4-*package 1.1-17; Bates et al., 2014) were carried out with R version 3.5.0 (R Core Team, 2018). To examine the level of multicollinearity the variance inflation factor (VIF) score for the final models were inspected. The rule of thumb cut-off criterion of 2.5 for deciding if a given independent variable displays too much multicollinearity was used (O'Brien, 2007). The VIFs of the predictors in models 1 and 2 were all <2.0, indicating that multicollinearity was not a problem. The R code is available online (https://osf.io/g39yk/) to reproduce all analyses.

## Results

### Individual CM and adult depressive symptoms

Individual scores of EM (*t* = 6.92, *p* < 0.001) and SA (*t* = 2.09, *p* = 0.037), but not PA (*t* = 1.25, *p* = 0.212) were significantly associated with the severity of depressive symptoms, see Table 2 for all model statistics. Of the covariates, being female (*t* = 2.99, *p* = 0.003) and having low education (*t* = *−*2.55, *p* = 0.011), but not age (*t* = 0.51, *p* = 0.610) were associated to depressive symptoms.

**Table 2.** Multilevel regression analyses on depressive symptoms: unconditional means model, baseline and individual model (*N* = 636)

	Unconditional means model						Baseline model						Individual model					
	Estimate	s.e.	*T*	*p*	95% CI		Estimate	s.e.	*T*	*p*	95% CI		Estimate	s.e.	*T*	*p*	95% CI	
Intercept	14.64	0.48	30.70	<0.001	13.70	15.57	14.56	3.05	4.79	<0.001	8.60	20.51	4.07	3.17	1.28	0.20	−2.15	10.29
EM													0.86	0.12	6.92	<0.001	0.62	1.10
PA													0.33	0.26	1.25	0.212	−0.19	0.84
SA													0.37	0.18	2.09	0.037	0.02	0.71
Covariates																		
Age							0.02	0.04	0.51	0.610	−0.05	0.09	−0.02	0.03	−0.57	0.567	−0.08	0.05
Educational level							−1.98	0.78	−2.55	0.011	−3.50	−0.46	−1.48	0.73	−2.02	0.043	−2.91	−0.05
Gender							2.49	0.83	2.99	0.003	0.80	4.07	1.64	0.79	2.06	0.039	0.08	3.19
Between-family variance IDS	21.28						20.97						17.06						
Within-family variance IDS	86.42						84.09						73.69						
ICC	0.20						0.20						0.19						
*F*							5.29						27.70						
*p*							0.001						<0.001						

EM, emotional maltreatment; PA, physical abuse; SA, sexual abuse.

Sex: 0 = male, 1 = female.

### Sibling concordance of CM types

To understand what types of CM are more family-wide and what types are more individual-specific, ICC were calculated. For EM the highest ICC (*r* = 0.37) was found, followed by PA (*r* = 0.21), indicating large and medium concordance among siblings, respectively (Donner et al., 1998). The ICC of SA was 0.04, which indicates almost no concordance of reports of SA within the family, meaning that siblings from the same family are not more alike in their reports of SA than random other individuals in the sample.

### Family levels and relative scores of CM with adult depressive symptoms

**Fig. 1.**
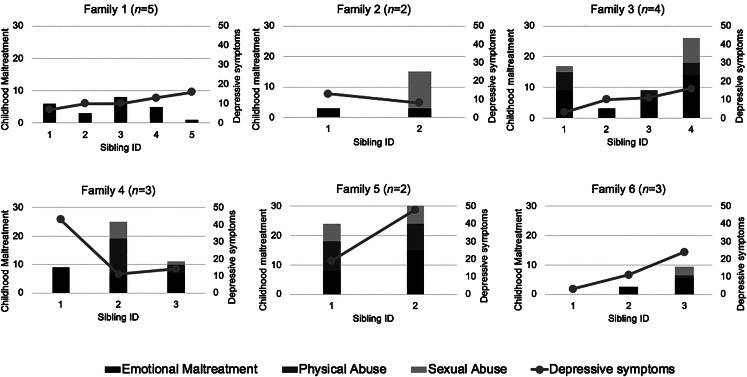
Childhood maltreatment (bars) and adult depressive symptoms (lines): An example of six random families from the sample

Figure 1 illustrates the variation between and within families in CM and depressive symptom levels in six families, randomly drawn from the study sample, emphasizing the value of this sibling design to provide more accurate estimates regarding the impact of CM on depressive symptoms. Whereas for example in family 1 no one reports PA or SA, in family 5 both individuals report moderate to severe EM, PA and SA, illustrating the differences between families. Within the families, some individuals report high levels of CM, whereas their sibling(s) do not, as is shown in families 2, 3 and 5.

To test whether the family level of CM related to depressive symptoms, model 1 contained the family means of EM, PA and SA and showed significant improvement in model fit compared to the baseline model including covariates only (*F*_(3, 36367)_ = 14.25, *p* < 0.001), see Table 3 for all model statistics. Compared to the baseline model, about 40% of the between-family variation in the IDS score was explained by the family levels of CM. The family mean of EM (*t* = 3.36, *p* < 0.001) and PA (*t* = 2.10, *p* = 0.036), predicted depressive symptoms, whereas for SA only a trend was found (*t* = 1.94, *p* = 0.052).

**Table 3.** Multilevel regression analyses on depressive symptoms: model 1 (family means of EM, PA and SA) and model 2 (relative EM, PA and SA) (*N* = 636)

	Model 1						Model 2					
	Estimate	s.e.	*T*	*p*	95% CI		Estimate	s.e.	*T*	*p*	95% CI	
Intercept	15.79	2.93	5.39	<0.001	10.05	21.53	15.79	2.86	5.52	<0.001	10.18	21.94
Emotional Maltreatment												
Family level	0.60	0.18	3.36	0.001	0.25	0.95	0.59	0.18	3.34	0.001	0.25	0.94
Relative level							1.12	0.18	6.40	<0.001	0.78	1.46
Physical Abuse												
Family level	0.89	0.42	2.10	0.036	0.06	1.71	0.91	0.42	2.17	0.030	0.09	1.73
Relative level							−0.01	0.33	−0.03	0.977	−0.65	0.63
Sexual Abuse												
Family level	0.60	0.31	1.94	0.052	−0.01	1.20	0.60	0.31	1.95	0.051	0.00	1.20
Relative level							0.19	0.22	0.90	0.370	−0.23	0.61
Covariates												
Age	−0.02	0.03	−0.46	0.643	−0.08	0.05	−0.01	0.03	−0.29	0.775	−0.07	0.06
Educational level	−1.55	0.75	−2.07	0.038	−3.02	−0.09	−1.35	0.73	−1.86	0.063	−2.78	0.07
Gender	2.08	0.81	2.55	0.011	0.48	3.67	1.74	0.79	2.21	0.027	0.20	3.28
Between family variance IDS		12.58						16.40				
Within-family variance IDS		83.14						73.18				
ICC		0.13						0.18				
*F*		14.25						16.18				
*P*		<0.001						<0.001				

*Note*: Sex: 0 = male, 1 = female.

In order to test the hypothesis whether being relatively more maltreated in comparison with the other siblings related to depressive symptoms, model 2 included the individual deviations from the family mean, i.e. relative scores, which was a significant improvement to model 1, i.e. family means only, *F*_(3, 42892)_ = 16.18, *p* < 0.001 (model 2). The relative EM score (*t* = 6.40, *p* < 0.001) was associated with depressive symptoms, whereas no association was found for relative PA score (*t* = −0.03, *p* = 0.977) and relative SA score (*t* = 0.90, *p* = 0.370). Compared to the baseline model, about 13% of the within-family variation in the IDS score was explained by the relative levels of CM.

### *Post hoc* specificity analyses

Anxiety disorders were highly prevalent in the current sample (53.1%); therefore, we tested for specificity of our findings to depressive symptoms by rerunning the analyses with anxiety symptoms as assessed by the Beck Anxiety Inventory (BAI; Beck, Epstein, Brown, and Steer, 1988). Individual scores of EM (*t* = 3.95, *p* < 0.001), PA (*t* = 2.11, *p* = 0.035) and SA (*t* = 2.29, *p* = 0.022) were significantly associated with the severity of anxiety symptoms, see Table A2 from Appendix 4 in the online Supplementary materials for all model statistics.

The family mean of PA (*t* = 2.14, *p* = 0.032) was associated with anxiety symptoms, whereas the family mean of EM (*t* = 1.83, *p* = 0.067) and SA (*t* = 1.83, *p* = 0.067) did not, see Table A3 from Appendix 4 in the online Supplementary materials for all model statistics. In line with our findings for depression, the relative EM score (*t* = 3.76, *p* < 0.001) was associated with anxiety symptoms, whereas no association was found for relative PA score (*t* = 0.93, *p* = 0.352) and relative SA score (*t* = 1.29, *p* = 0.196).

## Discussion

This sibling study on the impact of CM on adult depression examined (1) the extent to which reports on EM (abuse and neglect), PA and SA were similar among siblings (i.e. sibling concordance), and the association of (2a) the family level and (2b) the differential experience of EM, PA and SA of siblings (e.g. ‘being the black sheep’) with siblings' individual depressive symptoms in adulthood. In addition, associations with anxiety symptoms were tested to examine the specificity of our findings for depression.

### Sibling concordance of CM types

EM was most shared between siblings followed by PA, whereas SA was most individual specific, which was in line with our hypothesis and supporting previous studies (Bifulco et al., 1997; Hamilton-Giachritsis & Browne, 2005; Hines et al., 2006; Jean-Gilles & Crittenden, 1990; Witte et al., 2018). The levels of sibling concordance (i.e. highest for EM and lowest for SA) are in line with the findings that the majority of individuals reporting EM or PA indicated that a parent was the perpetrator, whereas the perpetrator of SA is often someone else rather than a first-degree relative (Hovens et al., 2009). The risk factors for EM described in the literature are mostly parental or environmental factors shared within a family (e.g. parental psychopathology and family structure), resulting in an increased risk of CM for all siblings. In contrast, risk factors for PA and SA appear to be predominantly individual characteristics (e.g. sex and child's behavioral problems, Hamilton-Giachritsis & Browne, 2005; Witte *et al*. 2018). To summarize, when an individual reports EM, there is an increased probability that a sibling experiences similar adversity. However, the other maltreatment types showed small to medium overlap within families (Donner et al., 1998), indicating that PA and SA experiences in childhood are quite unique to each individual of the family.

### CM in the family context and adult depressive symptoms

In line with earlier findings, our analyses showed that individual reports of EM and SA, but not PA, were simultaneously related to more depressive symptoms (Cecil et al., 2017; Infurna et al., 2016; Spinhoven et al., 2010). Results of our *post hoc* specificity analyses indicate that individual experiences of EM and SA are linked to elevated symptom levels of both depression and anxiety, whereas PA is only associated with anxiety, but not depression severity. However, when considerin*g* the family mean and relative level of CM a slightly different picture emerges. The family-wide effects of CM on an individual may be either direct, by being exposed to the same adverse parental behavior, or indirect, the so-called vicarious effect of CM (Spano, 2018). For EM, both the family means and relative levels (i.e. reporting more abuse or neglect than siblings) were jointly related to more depressive symptoms in adulthood. In addition to the family-wide impact, individuals who report more childhood EM than the family average also report more depressive symptoms. This may reflect the ‘Black Sheep Effect’. Previous studies in both children and adults indicate that differential parenting, e.g. the perception or actually receiving less affect and engagement from a parent than another child in the family (Boyle et al., 2004; Jenkins et al., 2016), are associated with negative outcomes such as depressive symptoms (Jensen et al., 2013; Meunier et al., 2012). Compared to EM, levels of reported PA varied more among siblings from the same family. Surprisingly, despite the variation of PA levels within the family, the family level of PA related to individual depression severity, whereas individual PA reports did not. It should be noted that, when CM types are examined in isolation, individual reports of PA do contribute to elevated depression levels. However, PA often occurs in the context of EM (Higgins & McCabe, 2001) and when modeled simultaneously, the effect of individual PA reports often diminishes or even disappears (Cecil et al., 2017; Spinhoven et al., 2010). In line with these observations, internalizing psychopathology is often mostly explained by emotional forms of abuse and neglect over and above PA (Cecil et al., 2017). In the case of EM, negative schemas and negative self-perceptions are explicitly handed to the child by parental criticism and belittlement, hence inducing a cognitive vulnerability for depressive symptomatology (van Harmelen et al., 2010; Wright, Crawford, & Del Castillo, 2009).

The family level's impact of physical violence on adult depression and anxiety, raises the question whether this is due to a vicarious effect by witnessing that a sibling has been abused or maltreated (Gerke et al., 2018), or whether reported physical violence is an indication of an adverse family environment in general, which in turn influences the development of later depressive and anxiety symptoms. The negative impact of the family level of EM and PA could thus also be an expression of a negative family atmosphere in which siblings grew up. Parental psychopathology, substance abuse or divorce, to which all family members are exposed, increase the risk of CM for all offspring (Witte et al., 2018) and also relate to adult depression (Lieb, Isensee, Höfler, Pfister, & Wittchen, 2002; Weissman et al., 2006). This interplay between family risk factors within the household should be further investigated. However, the simultaneous association between the family level of EM and PA with depression suggests that both types of CM are unique risk factors for depressive symptoms, rather than the same underlying construct like an overall negative family atmosphere. The association of the family level of PA with anxiety and depressive symptom levels suggests that physical violence has a common effect on adult anxiety and depression, whereas the family level of EM has a specific effect on depression as compared to anxiety. Although the individual report of SA was associated with depressive and anxiety symptoms, the family and relative levels of SA were not. This may be related to the fact that the perpetrator is usually someone outside the core family and the fact that if it occurs within the family it happens mostly in secret, which both may greatly reduce the chance of a vicarious effect. A methodological explanation of these findings is that the family mean of SA can be inflated due to one extreme value within the family while the other siblings report no SA. Therefore, association of the SA family mean with depression may reflect the association between an individual report of SA and depression rather than the overall family context. Associations of maltreatment types and depressive and anxiety symptoms remain when controlling for the main effects of gender and educational levels.

### Strengths and limitations

This is one of the few studies investigating multiple siblings per family, which contributes to the understanding of the family framework in which CM mostly occurs. Moreover, decomposing individual variables into a family level variable and relative scores helps us to differentiate between family-wide and individual-specific effects (Feaster et al., 2011; Jenkins et al., 2016). Another strength is the large clinically relevant sample, consisting of persons with a lifetime depressive or anxiety disorder and their affected and unaffected siblings. Considering the genetic background of depression (Smoller, 2016), it should be acknowledged that the study sample includes families with at least one affected family member, symptom levels in the sample may therefore be higher than in the general population. It should be noted, however, that PA and SA were less prevalent than EM which could have contributed to lower ICC for PA and SA. Nevertheless, despite the lower prevalence, PA and SA did contribute to depression in their unique way. Moreover, these numbers do seem to adequately reflect the sibling concordance for the three types of CM. Second, CM was measured using a retrospective instrument (the CTQ), which may be sensitive to recall bias. However, most studies indicate fairly good reliability of the CTQ (Hardt & Rutter, 2004) and are not critically affected by current mood disorders (Spinhoven et al., 2010). Previous studies have reported good test–retest reliability for the CTQ (Bernstein & Fink, 1998). Moreover, CTQ reports (W4) were highly correlated with reports on the Childhood Trauma Interview 4 years earlier (W1) in NESDA (Spinhoven et al., 2014), which indicates that the CTQ shows adequate consistency across time, suggesting stability of retrospective CM reports. Hence, it can be assumed that the difference of 5–8 years between data collection points of targets (W4) and siblings (W6) in retrospective recall does not significantly affect the reliability of the reports on childhood experiences. Third, the decomposed variables depend on the number of siblings participating within the family. That is, in large families an extreme score of an individual, as may be the case for SA, has a smaller effect on mean family level than in small families (Feaster et al., 2011). However, for EM and PA, i.e. abuse occurring within the household, differentiation between family and individual relative effects as risk factors for depression revealed new valuable information.

### Implications and future research

The overall family-wide experience (common across siblings) of EM and PA and the relative experience (individual difference from the family mean; the Black Sheep Effect) of EM contribute to adult depression levels. Our findings underline that the context of siblings' CM experiences play a vital role in an individuals' adult depression and anxiety. Even though the environmental influence of CM on depression is substantial, it is important to bear in mind that shared-genetic factors partly determine and explain the presence of depressive symptoms within families (Smoller, 2016). Moreover, our results provide a clear image of sibling concordance with respect to the three CM types, which helps to understand to what extent adult siblings share their (perception on) childhood experiences (see Plomin, 2011; Plomin & Daniels, 1987; Turkheimer & Waldron, 2000). Although siblings report similar levels of CM experiences, substantial differences within families also remain. Further research should focus on the underlying determinants of CM within the family to better understand the processes of parental maltreatment and how one individual from the same family may feel more or less depressed than a sibling after similar adverse childhood experiences. Future studies addressing the impact of childhood family adversities, should address the sibling context to disentangle the effect of the family system. Agreement between concurrent and retrospective reports of CM is low (Baldwin, Reuben, Newbury, & Danese, 2019). We therefore like to underline that our retrospective approach targets a specific group of adults reporting CM. Nevertheless, it should be acknowledged that both concurrent and retrospective reports of CM are linked to psychopathology (Newbury et al., 2018; Scott, McLaughlin, Smith, & Ellis, 2012). Observational and qualitative studies would be valuable to elucidate the within-family differences in more detail to identify individuals within the family at risk, and to advance the understanding of the influence of family context on adult depression. In clinical practice, health professionals should be aware of the effect of CM not only on the targeted individual, but also on other siblings. Informing parents, community health services, general practitioners and schools could lead to better identification and understanding of the impact of (parental) abuse and neglect and vulnerability to adult depression and anxiety. Moreover, focusing on the improvement of the family environment could potentially contribute to adequate prevention of both CM and psychopathology in long-term.

## Conclusion

Altogether, the findings of this study illustrate that the childhood experiences of adult siblings are in part interdependent and, furthermore, suggest that in addition to individual experiences, maltreatment experiences of a brother or sister may also have a long-term burden on an individual. Considering the experiences of multiple siblings from the same family and, moreover, differentiating between family and relative (within-family) level allows new insights into the individual and family-wide effects of CM on adult depression and anxiety.

## Supplementary material

For supplementary material accompanying this paper visit https://doi.org/10.1017/S0033291720000823. click here to view supplementary material
