# Supplementary material for: It is a family affair: individual experiences and sibling exposure to emotional, physical and sexual abuse and the impact on adult depressive symptoms
Source: Psychol Med. 2020 Apr 20;51(12):2063–73. doi: 10.1017/S0033291720000823 (PMC8426575; doi:10.1017/S0033291720000823)
Supplement: Supplementary file 1 [file S0033291720000823sup001.doc]

Appendix 1. Prevalence and distribution of emotional maltreatment (EM), physical (PA) and sexual abuse (SA) in the NESDA sibling sample

*Table A1*. Prevalence of EM, PA and SA

| Emotional maltreatment *M (SD*) | 9.85 (3.6) |
| --- | --- |
| no EM *N* (%) | 162 (25.5) |
| EM low  *N* (%) | 236 (37.1) |
| EM moderate  *N* (%) | 134 (21.1) |
| EM high  *N* (%) | 104 (16.4) |
| Physical abuse *M (SD*) | 5.63 (1.8) |
| No PA *N* (%) | 581 (91.4) |
| PA low  *N* (%) | 30 (4.7) |
| PA moderate  *N* (%) | 14 (2.2) |
| PA high  *N* (%) | 11 (1.7) |
| Sexual abuse *M (SD*) | 5.8 (2.4) |
| No SA *N* (%) | 521 (81.9) |
| SA low  *N* (%) | 54 (8.5) |
| SA moderate  *N* (%) | 39 (6.1) |
| SA high  *N* (%) | 22 (3.5) |


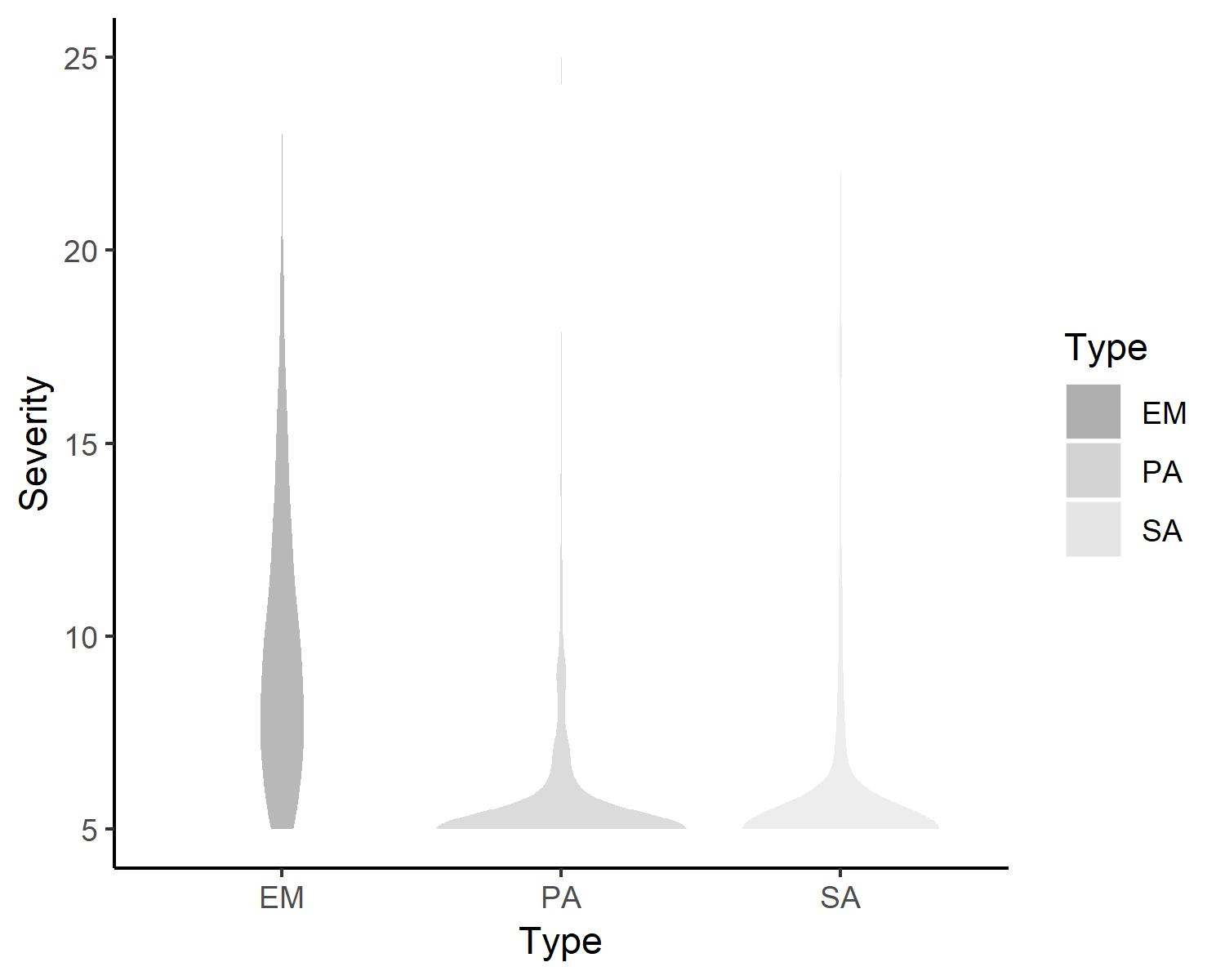


The violin plots in *Figure A1* represent the distribution of EM, PA and SA levels in the sample. Compared to EM, the density of low PA and SA scores is high. The levels of EM are more equally distributed as is also shown in table 1 of appendix 3.

*Figure A1.* Distribution of EM, PA and SA in the sample

Appendix 2. Multiple imputations of missing data

We generated 100 imputed datasets using 100 iterations each using multiple imputations (*mice-*package), carried out with R version 3.5.0 (R Core Team, 2018).. The imputation model and assessing the convergence of the algorithm was based on Vink & Van Buuren (2017a) and Grund, Robitzsch & Ludtke (2018). Because of the nested structure of the data we chose the *pan*-method (version 1.6, Schafer & Yucel, 2002, Grund, Ludtke, Robitzch, 2016) within the “multiple imputation by chained equations” method implemented in the *mice*- package (version 3.3.0, Van Buuren & Groothuis-Oudshoorn, 2011). To consider the fact participants are nested in families, a clustering variable, i.e. family number, indicating which siblings originate from the same family, was included in the imputation model. To estimate missing values on the IDS, data of earlier data collection waves (i.e. wave 1-5) on the IDS was added to the imputation model as predictor variable. Also, data of the NEMESIS interview (wave 1) was included in the model to estimate missing values on the CTQ. To evaluate the convergence of the imputations we examined trace lines and density plots, and compared means of the imputed datasets with the original dataset by visual inspection (Vink & Van Buuren, 2017b). The *mitml-*package (version 0.3-6) was used to combine the datasets for further analysis (Grund, Robitzsch & Ludtke 2016).

Appendix 3. Calculation of predictors

The two types of predictors were calculated as follows: The family means of maltreatment (model 1) were obtained by calculating the mean per family of each CTQ subscale of all siblings. For the analysis the family means were grand-mean centered. The relative scores (model 2) were calculated as each individual deviation from their family mean, also referred to as centering within context (CWC; Raudenbush & Bryk, 2002; Snijders & Bosker, 1999). With respect to the relative levels, positive scores indicate more reported CM compared to siblings of the same family and negative scores indicate less reported maltreatment compared to the siblings in the same family. Similar methods for decomposing family and individual relative effects from individual-level data were used in studies by Jenkins et al. (2009), Valgardson & Schwartz (2019) and Feaster et al. (2011)

## Appendix 4. Testing specificity: CM in the family context and adult anxiety symptoms

*Table A2.* Multilevel regression analyses on anxiety symptoms: unconditional means model, baseline and individual model (*N=*636)

|  | UNCONDITIONAL MEANS MODEL | | | | | | BASELINE MODEL | | | | | | | INDIVIDUAL MODEL | | | | | |  |
| --- | --- | --- | --- | --- | --- | --- | --- | --- | --- | --- | --- | --- | --- | --- | --- | --- | --- | --- | --- | --- |
|  | *Estimate* | *SE* | *T* | *p* | 95% CI | | *Estimate* | *SE* | *T* | *p* | 95% CI | | | *Estimate* | *SE* | *T* | *p* | 95% CI | |  |
| Intercept | 7.27 | 0.34 | 21.64 | <.001 | 6.62 | 7.93 | 6.27 | 2.21 | 2.84 | .004 | 1.95 | 10.59 | | -0.59 | 2.37 | -0.25 | 0.801 | -5.24 | 4.04 | |
|  |  |  |  |  |  |  |  |  |  |  |  |  | |  |  |  |  |  |  | |
| EM |  |  |  |  |  |  |  |  |  |  |  |  | | 0.38 | 0.10 | 3.95 | <.001 | 0.19 | 0.56 | |
| PA |  |  |  |  |  |  |  |  |  |  |  |  | | 0.41 | 0.20 | 2.11 | 0.035 | 0.03 | 0.79 | |
| SA |  |  |  |  |  |  |  |  |  |  |  |  | | 0.30 | 0.13 | 2.29 | 0.022 | 0.04 | 0.56 | |
| Covariates |  |  |  |  |  |  |  |  |  |  |  |  | |  |  |  |  |  |  | |
| Age |  |  |  |  |  |  | -0.01 | 0.03 | -0.59 | 0.558 | -0.06 | 0.03 | | -0.03 | 0.02 | -1.37 | 0.171 | -0.08 | 0.01 | |
| Educational level |  |  |  |  |  |  | -0.87 | 0.57 | -1.54 | 0.123 | -1.98 | 0.24 | | -0.58 | 0.55 | -1.06 | 0.287 | -1.65 | 0.49 | |
| Gender |  |  |  |  |  |  | 2.37 | 0.62 | 3.84 | <.001 | 1.16 | 3.57 | | 1.95 | 0.60 | 3.24 | 0.001 | 0.77 | 3.12 | |
| Between family variance IDS | 8.13 | | | | | | 7.50 | | | | | | 6.47 | | | | | | |  |
| Within family variance IDS | 49.39 | | | | | | 48.35 | | | | | | 44.85 | | | | | | |  |
| ICC | 0.14 | | | | | | 0.13 | | | | | | 0.13 | | | | | | |  |
| F |  | | | | | | 5.77 | | | | | | 16.04 | | | | | | |  |
| p |  | | | | | | 0.001 | | | | | | <.001 | | | | | | |  |

*Note.* Sex: 0 = Male 1 = Female, EM = Emotional Maltreatment, PA=Physical Abuse, SA=Sexual Abuse

*Table A3.* Multilevel regression analyses on anxiety symptoms: model 1 (family means of EM, PA and SA) and model 2 (relative EM, PA and SA) (*N* = 636)

|  | FAMILY MODEL 1 | | | | | | FAMILY MODEL2 | | | | | |  |
| --- | --- | --- | --- | --- | --- | --- | --- | --- | --- | --- | --- | --- | --- |
|  | *Estimate* | *SE* | *T* | *p* | *95% CI* | | *Estimate* | *SE* | *T* | *p* | *95% CI* | |  |
| Intercept | 6.88 | 2.17 | 3.17 | .002 | 2.63 | 11.14 | 6.73 | 2.15 | 3.14 | 0.002 | 2.53 | 10.93 |  |
|  |  |  |  |  |  |  |  |  |  |  |  |  |  |
| EM |  |  |  |  |  |  |  |  |  |  |  |  |  |
| family level | 0.24 | 0.13 | 1.83 | .067 | -0.02 | 0.50 | 0.24 | 0.13 | 1.82 | 0.069 | -0.02 | 0.49 |  |
| relative level |  |  |  |  |  |  | 0.51 | 0.14 | 3.76 | <.001 | 0.25 | 0.78 |  |
| PA |  |  |  |  |  |  |  |  |  |  |  |  |  |
| family level | 0.68 | 0.32 | 2.14 | 0.032 | 0.06 | 1.29 | 0.68 | 0.32 | 2.15 | 0.031 | 0.06 | 1.30 |  |
| relative level |  |  |  |  |  |  | 0.23 | 0.25 | 0.93 | 0.352 | -0.25 | 0.71 |  |
| SA |  |  |  |  |  |  |  |  |  |  |  |  |  |
| family level | 0.41 | 0.22 | 1.83 | .067 | -0.03 | 0.84 | 0.42 | 0.22 | 1.87 | 0.062 | -0.02 | 0.85 |  |
| relative level |  |  |  |  |  |  | 0.21 | 0.16 | 1.29 | 0.196 | -0.11 | 0.53 |  |
| Covariates |  |  |  |  |  |  |  |  |  |  |  |  |  |
| Age | -0.03 | 0.02 | -1.27 | 0.204 | -0.08 | 0.02 | -0.03 | 0.02 | -1.19 | 0.236 | -0.08 | 0.02 |  |
| Educational level | -0.68 | 0.56 | -1.23 | 0.218 | -1.77 | 0.40 | -0.51 | 0.55 | -0.93 | -.354 | -1.58 | 0.57 |  |
| Gender | 2.19 | 0.61 | 3.61 | <.001 | 1.00 | 3.38 | 1.96 | 0.60 | 3.27 | 0.001 | 0.79 | 3.13 |  |
| Between family variance IDS |  |  | 4.79 |  |  |  |  |  |  | 6.35 |  |  |  |
| Within family variance IDS |  |  | 48.30 |  |  |  |  |  |  | 44.65 |  |  |  |
| ICC |  |  | 0.09 |  |  |  |  |  |  | 0.12 |  |  |  |
| *F* |  |  | 8.51 |  |  |  |  |  |  | 8.96 |  |  |  |
| *p* |  |  | <.001 |  |  |  |  |  |  | <.001 |  |  |  |

*Note.* Sex: 0 = Male 1 = Female, EM = Emotional Maltreatment, PA=Physical Abuse, SA=Sexual Abuse.

**References**

Van Buuren, S., Groothuis-Oudshoorn, K. (2011). “mice: Multivariate Imputation by Chained Equations in R.” *Journal of Statistical Software*, 45(3), 1-67. Retrieved from: <https://www.jstatsoft.org/v45/i03/>.

Feaster, D., Brincks, A., Robbins, M., Szapocznik, J. (2011). Multilevel Models to Identify Contextual Effects on Individual Group Member Outcomes: A Family Example. *Family Process* **50**, 167–183.

Grund, S., Lüdtke O., & Robitzsch, A. (2018). Multiple imputation of missing data for multilevel models: Simulations and recommendations. *Organizational Research Methods*, *21*(1), 111-149.

Grund, S., Lüdtke., O., & Robitzsch, A. (2016). Multiple imputation of multilevel missing data: An introduction to the R Package pan. *SAGE Open*, *6*(4), 2158244016668220.

Grund, S, Robitzsch, A., Lüdtke, O. (2016). mitml: Tools for multiple imputation in multilevel modeling (Version 0.3-2). Retrieved from http://CRAN.R-project.org/package=mitml

Jenkins, J.M., Cheung, C., Frampton, K.L., Rasbash, J., Boyle, M.H., Georgiades, K. (2009). The use of multilevel modeling for the investigation of family process. *International Journal of Developmental Sciences* 3, 131–149.

R Core Team (2018). *R: A language and environment for statistical computing.* R Foundation for Statistical Computing, Vienna, Austria. URL: https://www.R-project.org/

Raudenbush, S.W., Bryk, A.S. (2002). *Hierarchical Linear Models: Applications and Data Analysis Methods - Stephen W. Raudenbush, Anthony S. Bryk - Google Boeken*. Sage.

Snijders, T.A., Bosker, R.J. (1999). *Multilevel Analysis: An Introduction to Basic and Advanced Multilevel Modeling*. Journal of the American Statistical Association: London.

Schafer, J.L., Yucel, R.M. (2002). Computational strategies for multivariate linear mixed-effects models with missing values. *Journal of computational and Graphical Statistics*, *11*(2), pp.437-457.

Valgardson, B.A., & Schwartz, J.A. (2019). An Examination of Within- and Between-Family Influences on the Intergenerational Transmission of Violence and Maltreatment. *Journal of Contemporary Criminal Justice* 35, 87–102.

Vink, G. & Van Buuren, S. (2017a). mice: Imputing multi-level data. Retrieved from: https://www.gerkovink.com/miceVignettes/Multi_level/Multi_level_data.html

Vink, G. & Van Buuren, S. (2017b). mice: Algorithmic convergence and inference pooling. Retrieved from: https://www.gerkovink.com/miceVignettes/Convergence_pooling/Convergence_and_pooling.html
